# Layered Metallic Vanadium Disulfide for Doubly Q-Switched Tm:YAP Laser with EOM: Experimental and Theoretical Investigations

**DOI:** 10.3390/nano11102605

**Published:** 2021-10-03

**Authors:** Ziqun Niu, Tianli Feng, Tao Li, Kejian Yang, Jia Zhao, Guiqiu Li, Dechun Li, Shengzhi Zhao, Wenchao Qiao, Hongwei Chu, Yizhou Liu, Kong Gao

**Affiliations:** 1 School of Information Science and Engineering, Shandong University, Qingdao 266237, China; 201712307@mail.sdu.edu.cn (Z.N.); litao@sdu.edu.cn (T.L.); zhaojia@sdu.edu.cn (J.Z.); gqiuli@sdu.edu.cn (G.L.); dechun@sdu.edu.cn (D.L.); wenchao.qiao@sdu.edu.cn (W.Q.); hongwei.chu@sdu.edu.cn (H.C.); yizhou.liu@sdu.edu.cn (Y.L.); 2 State Key Laboratory of Crystal Materials, Institute of Crystal Materials, Shandong University, Jinan 250100, China; k.j.yang@sdu.edu.cn; 3 Key Laboratory of Laser & Infrared System, Shandong University, Qingdao 266237, China; Kong93_G@163.com

**Keywords:** vanadium disulfide, doubly Q-switched laser, 2 μm wavelength region, pulse width, peak power

## Abstract

In the current study, layered metallic vanadium disulfide (VS_2_) is fabricated by a liquid-phase exfoliation method, and its microstructures as well as optical characteristics are investigated. Based on first-principles calculations, the band structure and density of the states of both bulk T-VS_2_ and monolayer H-VS_2_ are illustrated, showing the metallic behavior with a zero band gap. By using VS_2_ as the saturable absorber in a doubly Q-switched Tm:YAP laser with an EOM, the Q-switching laser pulses at 2 μm with 22 ns and 200 Hz are generated, corresponding to the single pulse energy of 755 μJ and the peak power of 34.3 kW. The coupled rate equations of the doubly Q-switched laser are given, and the numerical simulations agree with the experimental results. The results indicate that VS_2_ is a promising nanomaterial due to its nonlinear optical property. The doubly Q-switched laser demonstrates a high level of performance in reducing pulse width and enhancing pulse peak power.

## 1. Introduction

Over the past few decades, the eye-safe mid-infrared laser operating near the 2 μm wavelength region, emitted from thulium (Tm^3+^) or holmium (Ho^3+^) ion-doped materials, has attracted increasing interest in various fields, including material processing [1], remote sensing [2], medical procedures [3], and military applications [4]. For pulsed lasers in particular, the Q-switching, cavity dumping, and mode-locking techniques are widely used. Q-switching is an effective method for emitting energetic laser pulses for short periods of time, possessing wide-ranging possibilities in the application of solid-state lasers. In general, the active Q-switching operation can be realized with an acoustic-optical modulator (AOM) or an electro-optical modulator (EOM), in which the repetition rate can be controlled, and the pulse sequences are stable [5,6]. For passive Q-switching technology, the repetition rate of laser pulse is unstable, but it does possess a nonlinear saturable absorption mechanism. The double Q-switching operation, functioning with the cooperation of both active Q-switching and passive Q-switching, or two passive Q-switching operations, has been demonstrated as the effective technique in pulse width reduction, peak power enhancement, and symmetry improvement [7,8]. At the 2-μm wavelength region in particular, the doubly Q-switched lasers with active and passive Q-switching operations demonstrate obvious advantages for obtaining a more stable pulse, shorter pulse width and higher peak power [9,10,11,12].

As a saturable absorber (SA), many emerging graphene-like two-dimensional (2D) nanomaterials have been investigated in the 2 μm wavelength region [13,14], such as transition-metal dichalcogenides (TMDs) [15], g-C_3_N_4_ [16], MXenes [17], layered double hydroxide [18], and metal/covalent organic frameworks [19,20]. Among the TMDs, layered vanadium disulfide (VS_2_) exhibits outstanding synthetic properties [21,22]. Ultrathin VS_2_ exhibits promising ferromagnetic behavior, providing evidence for magnetic behavior in pristine monolayers without introducing defects or stress [23,24]. The large interlayer spacing and high surface activity of 2D VS_2_ render it suitable for application as an electrode material [25]. Moreover, VS_2_ is the softest candidate of the TMDs to date, revealing its inherent advantage in the application of nanomaterial-based flexible electronics [26]. The remarkable nonlinear optical responses of layered VS_2_ have already been reported in previous works [27,28]. In 2020, Li et al. demonstrated a passively Q-switched Er-doped fiber laser using VS_2_ SA with the shortest pulse width of 854 ns [27]. Pang et al. presented a passively mode-locking ultrafast fiber laser at 1.5 μm with pulse width of 169 fs [28]. However, as far as we know, both VS_2_-based pulsed lasers at 2 μm and doubly Q-switched lasers have not been reported in the literature.

In this work, we exploit the potential of VS_2_ nanosheets with a 2 μm pulsed laser, and a doubly Q-switched Tm:YAP laser based on the EOM and VS_2_ is presented. The morphology, microstructures, and optical characteristics of VS_2_ are comprehensively investigated. The first-principles-based density functional theory shows that VS_2_ is metallic with a zero band gap. At an absorbed pump power of 6.87 W, the shortest pulse of 22 ns at 200 Hz is obtained experimentally from the doubly Q-switched laser, corresponding to the peak power of 34.3 kW. In comparison with the singly Q-switched laser with VS_2_ or an EOM, the doubly Q-switched laser can generate a shorter pulse width and a higher peak power. The largest pulse width reduction ratio of 100 with a peak power enhancement factor of ~6000 is achieved. A rate-equation model is built to explore the Q-switching mechanism, and the numerical simulations agree with the experimental results. Our research suggests that the layered VS_2_ has a good modulation effect on optoelectronic applications.

## 2. Fabrication and Characterization of 2D VS_2_ SA

The liquid-phase exfoliation method was employed to fabricate VS_2_ nanosheets from its bulk counterparts. Considering that N-methyl pyrrolidone (NMP) is demonstrated to be a good solvent with suitable surface energy for exfoliating TMD materials [29], we dissolved the 10 mg VS_2_ powder into a 10 mL NMP solvent in the fabrication. The resultant dispersion was ultrasonically treated in cold water for 2 h, and followed by a centrifuging operation at 6000 rpm for 10 min. During this process, the VS_2_ nanosheets with different thicknesses were distributed in different layers in the solution, and the supernatant was taken out and then spin-coated on the glass substrate. After the drying process, a layered VS_2_ nanosheets-based SA was obtained.

**Figure 1:**
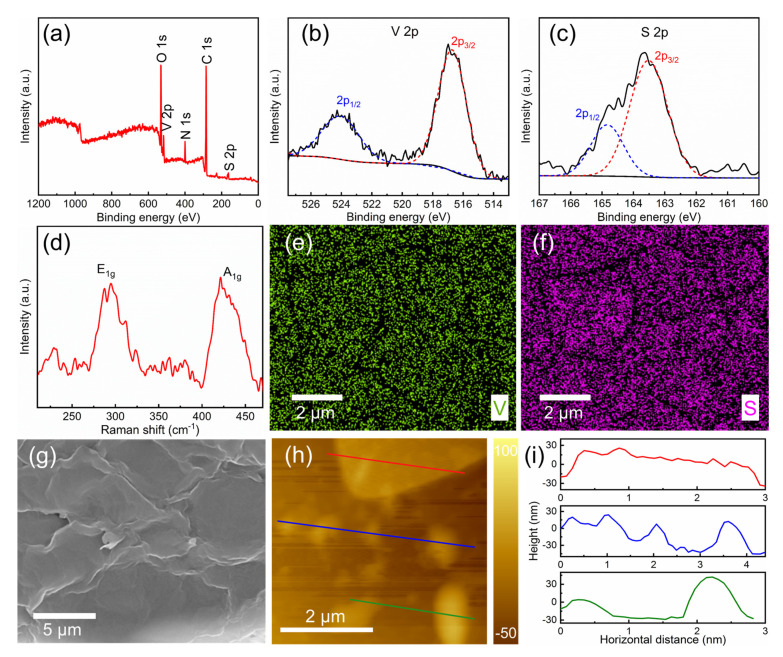
(**a**) XPS survey spectrum and core high-resolution spectra of (**b**) V 2p; (**c**) S 2p for the VS_2_ nanosheets; (**d**) Raman spectra of VS_2_ nanosheets; (**e**,**f**) EDS images showing morphology and element distribution; (**g**) SEM image of VS_2_ samples; (**h**) AFM image; and (**i**) the corresponding height profile of VS_2_ SA.

The characterization results of the liquid-phase exfoliated 2D VS_2_ nanosheets are shown in Figure 1. XPS analysis of the VS_2_ nanosheets was used to determine the bonding characteristics and the composition. Besides the V and S elements, the C, O, and N elements are detected in Figure 1a, which may be caused by residual NMP. The XPS peaks of 2p_1/2_ and 2p_3/2_ orbitals of V and S ions are shown in Figure 1b,c [24,30]. The peaks located at 517 and 524 indicate the V^4+^ chemical state, which confirms the presence of VS_2_ [31]. Raman spectroscopy was investigated using a LabRAM HR Evolution spectrometer (Horiba, France). The 532 nm laser was irradiated on the as-prepared nanosheets via a 50× objective lens, and the Raman shifts ranging from 210 to 470 cm^−1^ are displayed in Figure 1d. Two typical active modes of E_1g_ and A_1g_, which correspond to in-plane and out-of-plane vibrations of VS_2_, respectively, were observed [32]. According to the energy dispersive spectrometer (EDS) mapping in Figure 1e,f, the V and S elements evidently spread over the surface of the exfoliated VS_2_ nanosheets. For SEM measurements in Figure 1g, the VS_2_ samples have an estimated lateral size of less than 10 μm, layering on top of each other. Figure 1h,i exhibit the atomic force microscopy (AFM) image and the corresponding height profile, which demonstrates that VS_2_ topography is assembled by a series of nanosheets with a thickness of 10–70 nm, suggesting a high uniformity of thickness.

**Figure 2:**
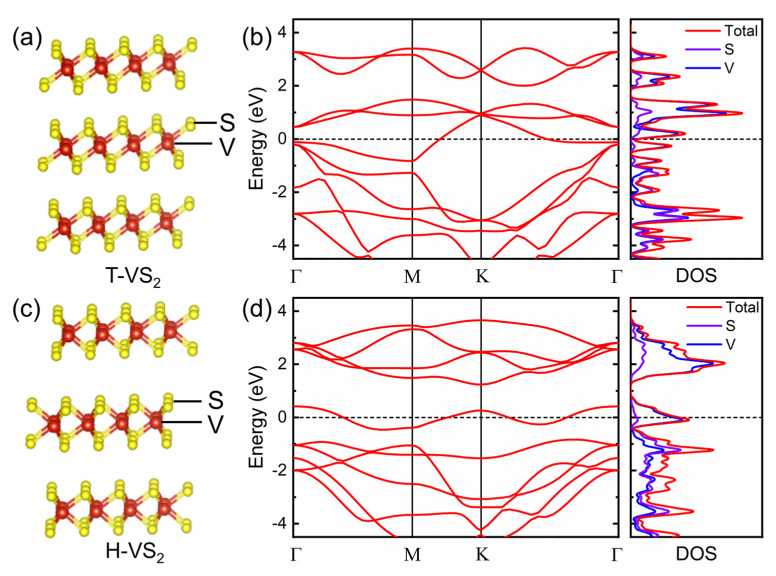
Lattice structure of (**a**) T-VS_2_ and (**c**) H-VS_2_; band structure diagrams and DOS of (**b**) bulk T-VS_2_ and (**d**) monolayer H-VS_2_. The dotted black line indicates Fermi energy, which is assigned a value of zero.

VS_2_ possesses two distinct polymorphs of trigonal (T, D3d point group, Figure 2a) and hexagonal (H, D3h point group, Figure 2c) phases, which are greatly affected by the nanosheet thickness and temperature [25]. Previous research has predicted that the bulk T-VS_2_ is more stable at room temperature but will transform to the H phase as the thickness decreases [33]. The structure of a monolayer VS_2_ consists of one V layer sandwiched between two S layers, and the VS_2_ layers are stacked together by weak van der Waals interactions with a spacing of 5.76 Å [34]. To describe the band structure and density of states (DOS) of VS_2_, we conducted first-principles-based density functional theory computations with the Perdew–Burke–Ernzerhof (PBE) generalized gradient approximation (GGA) functional. The high-symmetry points Γ, M, and K in the band structure diagrams are defined as the center, edge midpoint, and corner of the Brillouin zone, respectively. As illustrated in Figure 2b,d, both bulk T-VS_2_ and monolayer H-VS_2_ show either metallic behavior or a zero band gap with a considerable total DOS across the Fermi level, indicating high microscopic 2D conductivity [35]. The partial DOS demonstrates that both V-3d and S-3p states are attributed to the metallic states.

**Figure 3:**
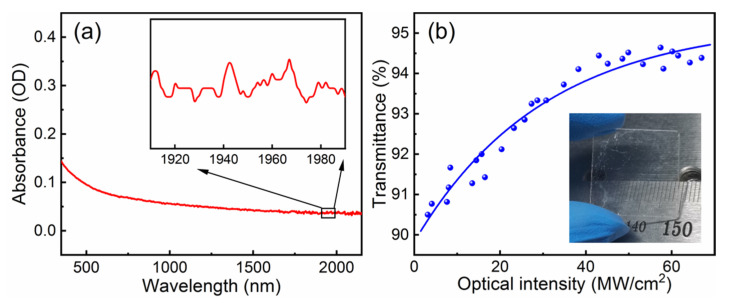
Optical characteristics of VS_2_. (**a**) The linear absorbance of the VS_2_ nanosheets; (**b**) the nonlinear optical transmittance curve of the VS_2_ SA. The inset of (**b**) presents a VS_2_ film deposited on a glass substrate.

The linear optical characteristic of the as-obtained VS_2_ nanosheets was obtained by using a UV–Vis–NIR spectrophotometer, as illustrated in Figure 3a. VS_2_ exhibited a broad absorption range covering the visible to mid-infrared wavelength region, and the detail data near 2 μm are provided in the inset of Figure 3a. The nonlinear optical feature of VS_2_ film on the glass substrate was examined via a balanced twin detector measurement over a long period. The Q-switched laser emitting at 2 μm acts as the optical source, with a repetition rate of 500 Hz and a pulse width of 50 ns. The optical intensity is equal to the peak power of the laser pulse divided by the effective area of the laser beam. The experimental data of VS_2_ SA can be fitted by the following equation:

(1) $$T=1-\alpha_{NS}-\frac{\Delta R}{1+I/I_{sat}},$$

where the non-saturable absorption loss *α_NS_* is estimated to be 5%. The modulation depth Δ*R* and the saturated optical intensity *I_sat_* are approximately 5% and 30 MW/cm^2^, respectively.

## 3. Experimental Setup

**Figure 4:**
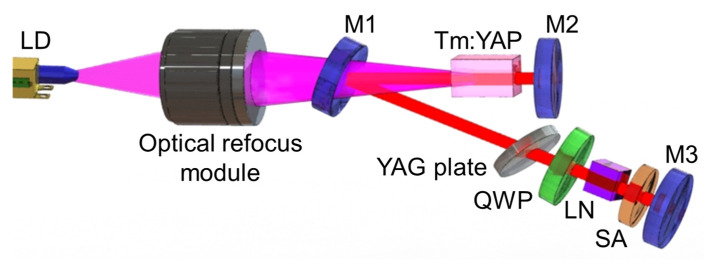
Schematic illustration of the Tm:YAP laser in double Q-switching operation.

The experimental setup of a doubly Q-switched laser with the VS_2_ SA and EOM is shown in Figure 4, in which a folded V-shaped cavity with a physical length of 160 mm is selected. Using this resonant cavity, the mode-matching condition can be satisfied, and the effect on the SA created by the pump laser is eliminated. The pumping source in the experiment is a commercial fiber-coupled LD (Focuslight, Xi′an, China), and the laser beam radiates at 792 nm. The pump beam is focused into the gain medium using an optical refocus module, about 200 μm in diameter. The a-cut Tm:YAP crystal is 3 at.% Tm^3+^ ion doped with dimensions of 3 × 3 × 10 mm^3^, and it is water cooled at 14 °C for heat dissipation. The absorption efficiency of the pumping beam in the Tm:YAP crystal is about 75%. The concave mirror M1 (Radius of curvature: 100 mm) is anti-reflection coated at 792 nm and high-reflection coated at 1.85–2.15 μm. The plane mirror M2 possesses the same coating as M1. The plane output mirror M3 has a transmission of 8% at 2 μm. The distances of M1 to M2 and M1 to M3 are 65 and 95 mm, respectively. The EOM configuration consists of the polarizer (YAG plate placed with Brewster angle), quarter-wave (λ/4) plate (QWP), and LiNbO_3_ (LN) crystal. The voltage on the LN is supplied by a QBU-BT Pockels cell driver with a working mode of the pulse-on scheme (normally off). The EOM and SA control the active and passive Q-switching operations separately, and the continuous-wave (CW) operation is achieved without an EOM or SA. The laser output is monitored by a laser power meter (EPM1000, Coherent) and an optical spectrometer (waveScan, APE). The temporal pulse characteristics are collected by an InGaAs detector (ET-5000, EOT) and recorded by an oscilloscope (DPO7104C, Tektronix).

## 4. Experimental Results

**Figure 5:**
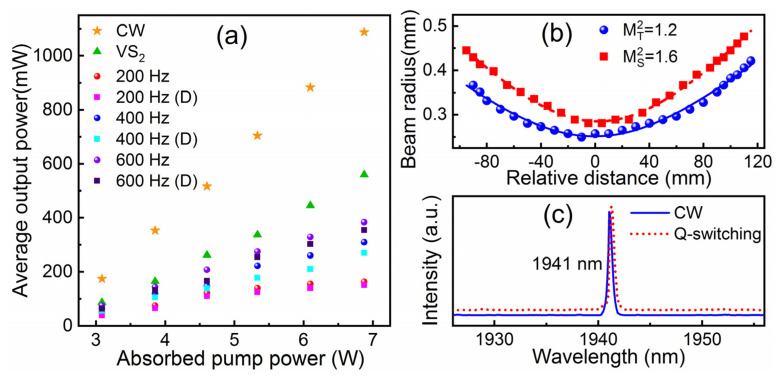
(**a**) Average output power, (**b**) M^2^ factors, and (**c**) emission spectra of CW and Q-switched lasers. D—doubly Q-switched laser.

The output power of CW laser and the average output powers of the Q-switched lasers versus pump power are exhibited in Figure 5a, showing an almost linear trend. The maximum output power of 1.09 W is obtained in a free-generation regime for 6.87 W absorbed pump power, corresponding to an optical-to-optical efficiency of 16%, the laser operated with 24% slope efficiency. To protect the VS_2_ SA and LN crystal, the absorbed pump power is not increased to above 6.87 W. The output power of Q-switched lasers is lower than that of CW operation regimes due to the insertion loss of the modulation device, and the output power of the doubly Q-switched laser is the lowest. Furthermore, the lower modulation rate means a lower energy utilization efficiency. The average output power of doubly Q-switched lasers at 200/400/600 Hz is 151/270/355 mW. The repetition rate is not increased further, considering the piezoelectric ringing effect in the LiNbO_3_ crystal. The quality factor (M^2^) of the solid-state laser is recorded by the knife-edge method. In Figure 5b, the M^2^ factor is measured for the doubly Q-switch operating mode under maximum output power. The M^2^ values are fitted to be 1.2 in the T direction and 1.6 in the S direction. The difference is attributed to the V-shaped folding of the resonant cavity. The central emission wavelengths of CW and Q-switched lasers are all located at 1941 nm with a linewidth at FWHM of ~0.5 nm, as shown in Figure 5c. The absorbed pump power and the modulated frequency rate have little effect on wavelength shifts in this experiment.

**Figure 6:**
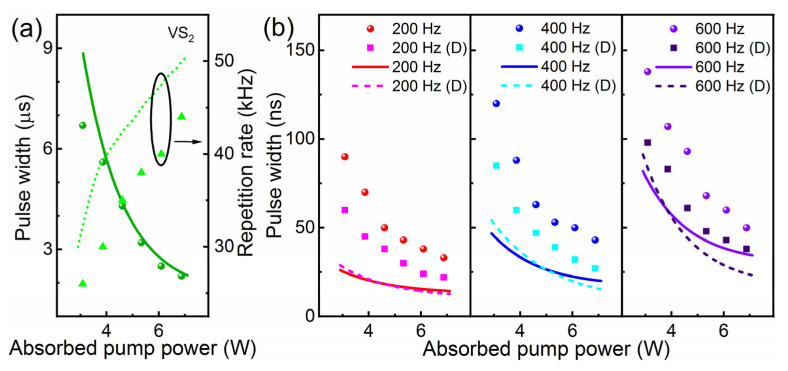
Relationship of absorbed pump power and pulse width (repetition rate) of (**a**) passively, (**b**) actively and doubly Q-switched lasers: symbols—experimental values, curves—simulated results.

The pulse characteristics, including pulse width and repetition rate, are exhibited in Figure 6 (symbols). With the absorbed pump power increasing from 3.08 to 6.87 W, the pulse repetition rate of a passively Q-switched laser is 26–44 kHz. The repetition rates of actively Q-switched and doubly Q-switched lasers are in accordance with the modulation rate of the EOM. Fixing the absorbed pump power at 6.87 W, the shortest pulse duration of 22 ns is measured in a doubly Q-switched laser operating at 200 Hz. The pulse width increases with the repetition rate from 200 to 600 Hz. However, the pulse widths of the doubly Q-switched laser are always shorter than those of the singly Q-switched laser.

**Figure 7:**
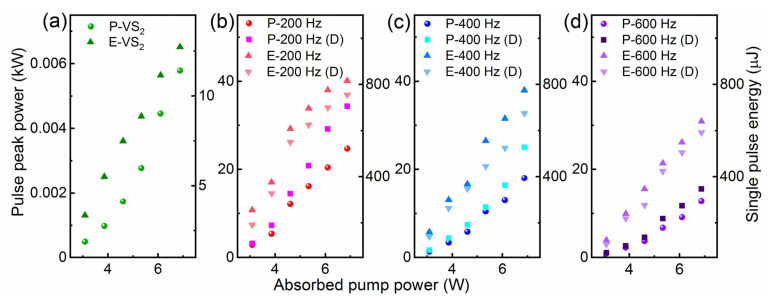
Relationship between absorbed pulse peak power, single pulse energy, and pump power of (**a**) VS_2_ Q-switched laser, (**b**) actively and doubly Q-switched lasers at 200 Hz, (**c**) actively and doubly Q-switched lasers at 400 Hz, (**d**) actively and doubly Q-switched lasers at 600 Hz. P—peak power; E—pulse energy.

The pulse peak power for passively, actively, and doubly Q-switched lasers are displayed in Figure 7. The doubly Q-switched laser generates higher peak power than that of a singly Q-switched laser. At the maximum absorbed pump power of 6.87 W, the pulse peak power is measured to be 5.8 W for the passively Q-switched laser, 24.7/18.0/12.8 kW for the EOM Q-switched laser at 200/400/600 Hz, and 34.3/25.0/15.6 kW for the doubly Q-switched laser at 200/400/600 Hz. The pulse peak power continuously increases with the absorbed pump power. The single pulse energy is also displayed in Figure 7, which is calculated through dividing average output power by the repetition rate. At the maximum pump power, the corresponding single pulse energies are 12.7 μJ, 816/775/640 μJ, and 755/675/592 μJ.

**Figure 8:**
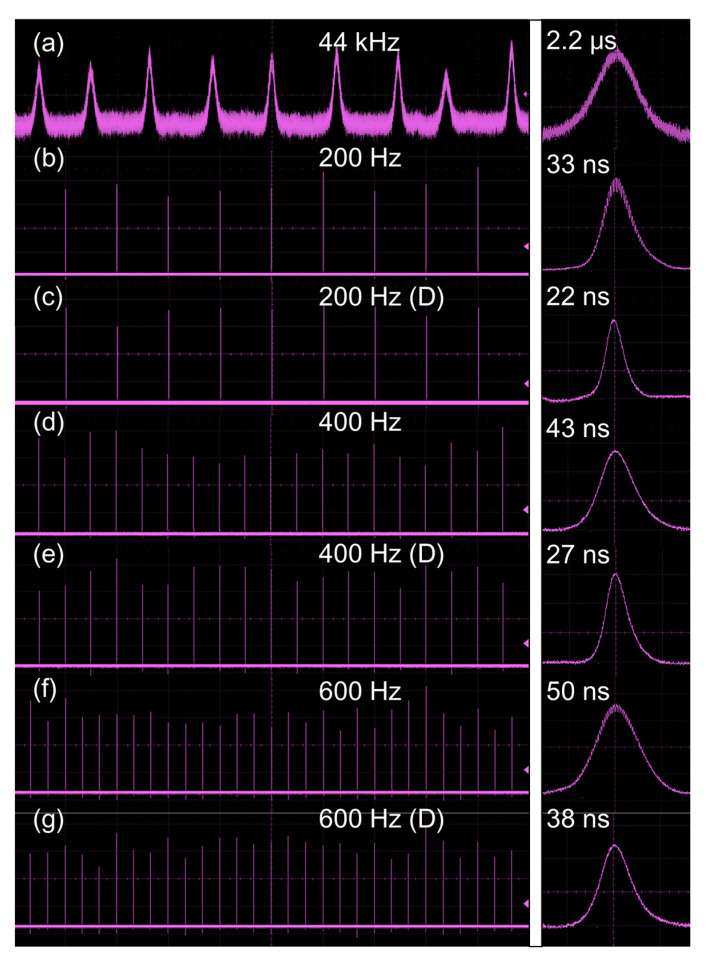
Q-switched temporal pulse sequences and single pulse profiles at pump power of 6.87 W. (**a**) VS_2_ Q-switched laser, (**b**,**d**,**f**) EOM Q-switched lasers at 200, 400, and 600 Hz, (**c**,**e**,**g**) doubly Q-switched lasers at 200, 400, and 600 Hz.

Figure 8 shows the typical temporal pulse sequences and single pulse profiles at a pump power of 6.87 W. One can see that doubly Q-switched lasers are more stable than singly Q-switched lasers. The amplitude fluctuations of pulse trains are ~16%/21%/23% for actively Q-switched lasers at 200/400/600 Hz, ~25% for passively Q-switched lasers at 44 kHz, and ~11%/15%/21% for doubly Q-switched lasers at 200/400/600 Hz, respectively. A series of shorter-duration pulses are generated in the doubly Q-switched lasers. To evaluate the pulse width reduction and peak power improvement, we define a reduction ratio (*t_c_*) and an improvement factor (*P_i_*):

(2) $$t_{c}=t_{s}/t_{d},$$

(3) $$P_{i}=P_{d}/P_{s},$$

where *t_s_* and *t_d_* represent the pulse widths of singly and doubly Q-switched lasers, and *P_s_* and *P_d_* are the peak powers, respectively. In comparison to the passively Q-switched laser, the *t_c_* values are 100.0, 81.5, and 57.9 at 200, 400, and 600 Hz under the pump power of 6.87 W, corresponding to the *P_i_* of 5932, 4321, and 2691, respectively. For actively Q-switched lasers at 200, 400, and 600 Hz, the *t_c_* values are 1.5, 1.6 and 1.3, corresponding to the *P_i_* of 1.39, 1.39 and 1.22, respectively. The shortest pulse width and the highest peak power are obtained at a low repetition rate (near the lifetime of the upper laser level).

## 5. Theoretical Analysis

**Figure 9:**
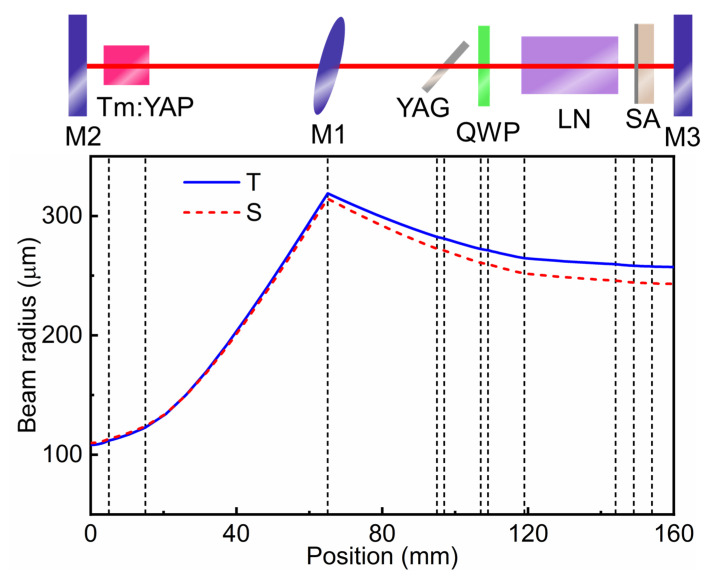
Laser beam radius distribution along the propagation direction.

According to the ABCD transmission matrix theory, the beam radius of the Tm:YAP laser along the propagation direction of an experimental setup is numerically simulated and depicted in Figure 9. The thermal lens effect is simplified with a focal length of 100 mm. The beam radius at the surface of the Tm:YAP crystal facing towards the M1 is calculated to be ~120 μm, similar to the size of the incident pump laser beam. The laser radius at VS_2_ SA is estimated as 250 μm. The angle of two arms in the V-shaped cavity makes a difference to the laser radius distribution in tangential (T) and sagittal (S) directions.

We apply a transverse electric field on the LN crystal in our experiment. The electric field is along the x- or y-axis and the laser propagate along the z-axis so that the voltage can be reduced, in comparison with the longitudinal electro-optical modulation with a high electric field. It is worth mentioning that the low voltage and pulse-on mode can extend the lifetime of the EOM, considering the electrochemical degradation effects [36]. The λ/4 voltage of the LN crystal is expressed as follows [37]:

(4) $$V_{\lambda /4}=\frac{\lambda d}{4n_{0}^{3}\gamma_{22}l},$$

where the ordinary refractive index *n*_0_ = 2.193, the electro-optic coefficient *γ*_22_ = 6.8 pm/V, *d* is the thickness, and *l* is the length of LN crystal. The λ/4 voltage is calculated to be 2.43 kV, concerning the 9 × 9 × 25 mm^3^ LN crystal. The modulation loss caused by the EOM can be written as:

(5) $$\delta_{EOM}=\cos^{2}\left(\frac{\pi }{2}\frac{V}{V_{\lambda /4}}\right)+\delta_{0},$$

where the initial loss of the EOM *δ*_0_ is approximately 3%.

To understand the Q-switching mechanism based on the SA and EOM, a coupled rate-equation model with plane wave approximation is proposed, as follows [12,38,39,40,41]:

(6) $$\frac{d\phi }{dt}=\frac{\phi }{t_{r}}[2n\sigma_{se}l_{YAP}-\ln\left(\frac{1}{1-T_{OC}}\right)-L-2\delta \left(\phi \right)-\delta_{EOM}]+\frac{n}{\tau }\frac{2\Omega_{L}}{4\pi },$$

(7) $$\frac{dn}{dt}=\left(1+\frac{f_{1}}{f_{2}}\right)\frac{\eta_{B}\eta_{p}P_{abs}}{h\nu_{p}\pi \omega_{p}^{2}l_{YAP}}\left(1-\frac{n}{N_{Tm}}\right)-\left(1+\frac{f_{1}}{f_{2}}\right)\sigma_{se}\frac{c}{n_{YAP}}\phi n-\frac{n}{\tau },$$

where *ϕ* is the intra-cavity photon density, *n* is the population-inversion density, *t_r_* is the round-trip time, *δ* (*ϕ*) represents laser intensity-dependent absorption of the SA (*δ* (*ϕ*) = 1 − *T* (*ϕ*)), *P_abs_* is the absorbed pump power, *ν_p_* is the frequency of the pump laser, *h* is Planck constant, and *c* is the velocity of light. The last item in Equation (4) means spontaneous radiation, where Ω*_L_* is the space angle of laser irradiating in both forward and backward directions [39]. The *f_1_* (~0.0284) and *f_2_* (~0.2544) are the Boltzmann thermal occupation factors of the lower and upper laser levels. The other parameter information is listed in Table 1. The single active Q-switching or passive Q-switching can be described when removing *δ* (*ϕ*) or *δ_EOM_*, respectively.

**Figure 10:**
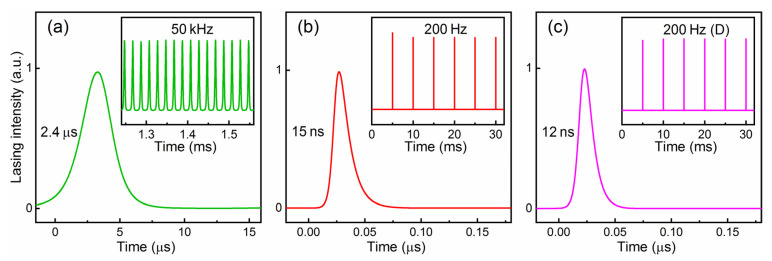
Simulated Q-switched single pulse profiles and temporal pulse sequences (inset). (**a**) Passive Q-switching, (**b**) active Q-switching, and (**c**) double Q-switching.

Through a numerical solution, the simulated sequential pulses and single pulse profiles based on Equations (4) and (5) for the passively, actively (200 Hz), and doubly (200 Hz) Q-switched lasers are presented in Figure 10. As the persistent pump builds up, the population of the ^3^F_4_ energy level continuously accumulates under modulation. The oscillation of the 2 μm laser based on the ^3^F_4_ to ^3^H_6_ transition will be generated once the net round-trip gain in the cavity becomes positive. The emitting of the laser is accompanied with the depletion of the population inversion. The simulated results display stable equal-interval pulse trains in the time domain. The rapidly falling edge of the passive Q-switching and the sharply rising edge of the active Q-switching may both act on the narrow pulse of the double Q-switching. The nonlinear effects, such as two-photon absorption and free-carrier absorption in giant pulses, can promote the depletion of population inversion [45].

The pulse width and the repetition rate for singly and doubly Q-switched lasers are calculated and shown in Figure 6 with curves. The theoretical solutions are basically consistent with the experimental results. The pulse width decreases while the repetition rate of the passively Q-switched laser increases with the increase in absorbed pump power. Under high pump power, the pulse width of the doubly Q-switched laser is the shortest. We believe that a more accurate result may be acquired with more precise values of related parameters and the intensity distribution of the laser beam.

This work shows the superior performance of a double Q-switching operation in reducing pulse width and enhancing peak power. The pulse characteristics of Q-switched lasers are described and analyzed experimentally and theoretically. The pulse width may be further reduced by adjusting nonlinear optical parameters of the SA, and the peak power may be enhanced. The defects, doping, surface or edge functionalization, and van der Waals interactions engineering may become useful at a later stage [46,47]. The experimental and theoretical explorations present an opportunity for 2D materials to be applied in the nonlinear optics field.

## 6. Conclusions

In summary, we report here, for the first time, the optical modulation features of layered metallic VS_2_ for Q-switching at a 2 μm wavelength region. The liquid-phase exfoliation method is successfully employed to fabricate VS_2_ nanosheets, and the microstructure characterizations are investigated. The band structure and DOS are calculated according to the first-principles-based density functional theory. Based on the VS_2_ SA and EOM, a double Q-switching operation for the Tm:YAP laser is realized. At an absorbed pump power of 6.87 W, the average output power, shortest pulse width, repetition rate, single pulse energy, and peak power are 151 mW, 22 ns, 200 Hz, 755 μJ and 34.3 kW, respectively. The experimental results display the advantage of double Q-switching. In comparison with the singly Q-switched laser, the pulse width of the doubly Q-switched laser is significantly reduced, and the peak power is efficiently improved. A rate-equation model is proposed to describe the Q-switching process, and the numerical solutions of the equations are in agreement with the experimental results. We believe that the VS_2_ nanomaterial possesses a promising opportunity for use in advanced optoelectronic devices.

## Figures and Tables

**Table 1:** Partial parameter of numerical modeling [40,41,42,43,44].

Parameter	Notation	Value
Emission cross-section	*σ_se_*	2.3 × 10^−21^ cm^2^
Length of Tm:YAP crystal	*l_YAP_*	10 mm
Intra-cavity loss	*L*	5%
Upper laser level lifetime	*τ*	4 ms
Pump quantum efficiency	*η_p_*	1.54
Beam overlap efficiency	*η_B_*	0.5
Tm^3+^ dopant concentration	*N_Tm_*	5.9 × 10^20^ cm^−3^
Refractive index	*n_YAP_*	1.92
Radius of pump laser	*ω_p_*	100 μm
Output transmittance	*T_OC_*	8%

## Data Availability

The data presented in this study are available in this article.
